# Comparative Study on Planetary Magnetosphere in the Solar System

**DOI:** 10.3390/s20061673

**Published:** 2020-03-17

**Authors:** Ching-Ming Lai, Jean-Fu Kiang

**Affiliations:** Graduate Institute of Communication Engineering, National Taiwan University, Taipei 10617, Taiwan; r06942015@ntu.edu.tw

**Keywords:** simulation, magnetosphere, magnetohydrodynamics, solar wind, Mercury, Earth, Jupiter, Uranus

## Abstract

The magnetospheric responses to solar wind of Mercury, Earth, Jupiter and Uranus are compared via magnetohydrodynamic (MHD) simulations. The tilt angle of each planetary field and the polarity of solar wind are also considered. Magnetic reconnection is illustrated and explicated with the interaction between the magnetic field distributions of the solar wind and the magnetosphere.

## 1. Introduction

Solar wind is a plasma stream ejected outwards from the solar corona. As solar wind blows near the Earth, the geomagnetic field is compressed on the dayside and elongated on the nightside, detouring the solar wind from directly reaching the Earth surface [1]. The magnetopause takes shape where the ram pressure of solar wind is balanced by the geomagnetic-field pressure [2]. A bow shock emerges upstream of the magnetopause to impede the supersonic solar-wind, forming the magnetosheath between the bow shock and the magnetopause [3].

The observation data, usually collected with satellites, are very sparse as far as the vast volume of magnetosphere is concerned. In addition, non-uniform temporal variation and inhomogeneous spatial distribution of solar wind make real-time measurement in global scale very difficult. Magnetohydrodynamic (MHD) simulations can provide a more holistic picture to study the interaction between solar wind and planetary magnetosphere [4,5,6]. More physical insights can be gained by systematically comparing the magnetosphere of different planets in our solar system [7]. For example, Mercury carries a relatively small magnetosphere due to its weak magnetic field. The magnetospheres of Jupiter and Saturn are dominated by fast rotation and internal plasma provided by Io and Enceladus, respectively. Jupiter carries the strongest magnetic field among all planets in the solar system. The volcanic activities on Io provides abundant sulfur dioxide (SO${}_{2}$) in its atmosphere, which are constantly ejected into the Jovian magnetosphere, forming neutral cloud around Io orbit, which is ionized by collisions, pumping plasma stream to the Jovian magnetosphere [8]. The magnetosphere of Uranus switches between an open configuration and a closed one in each Uranus day (about 17.24 h) [9] due to its large tilt angle of magnetic-dipole axis and subsequent asymmetric bow-shock [10].

Magnetic reconnection is generally caused by two crashing magnetic fields with opposite polarities, leading to a narrow region of low magnetic-field intensity where these two fields meet. It is accompanied by the conversion of magnetic energy to kinetic energy of particles. In this work, MHD simulations are conducted to compare the magnetospheric responses of Mercury, Earth, Jupiter and Uranus, under northward and southward interplanetary magnetic fields (IMFs), separately. The occurrence and features of magnetic reconnection under different IMFs and dipole orientations are also systematically compared in this work. The rest of this work is organized as follows. A model of solar wind–magnetosphere coupling is presented in Section 2, simulation results on magnetospheres of these four planets are presented and elaborated in Section 3, Section 4, Section 5 and Section 6, respectively, followed by some conclusions drawn in Section 7.

## 2. Model of Solar Wind–Magnetosphere Coupling

The MHD equations for ideal plasma are [11]

$$\begin{array}{c} \;\frac{\partial \rho }{\partial t}+\nabla \cdot \left(\rho \bar{u}\right)=0\\ \;\frac{\partial (\rho \bar{u})}{\partial t}+\nabla \cdot \left(\rho \bar{u}\bar{u}+P_{t}\bar{\bar{I}}-\frac{\bar{B}\bar{B}}{\mu_{0}}\right)=0\\ \;\frac{\partial \bar{B}}{\partial t}-\nabla \times \left(\bar{u}\times \bar{B}\right)=0\\ \;\frac{\partial e}{\partial t}+\nabla \cdot \left[\left(e+P_{t}\right)\bar{u}-\frac{\bar{B}\bar{B}\cdot \bar{u}}{\mu_{0}}\right]=0\\ \;\nabla \cdot \bar{B}=0 \end{array}$$

where $\rho =\sum_{\sigma }m_{\sigma }n_{\sigma }$ is the total mass density, with σ=e for electrons and *i* for ions, $m_{\sigma }$ and $n_{\sigma }$ are the particle mass and number density, respectively, of species σ; $\bar{u}$ is the velocity, $\bar{B}$ is the magnetic field, $P_{t}=P+B^{2}/\left(2\mu_{0}\right)$ is the total pressure, *P* is the gas pressure, $\mu_{0}$ is the permeability in free space, $\bar{\bar{I}}$ is an identity tensor, $e=\rho u^{2}/2+P/\left(\gamma -1\right)+B^{2}/\left(2\mu_{0}\right)$ is the total energy density and $\gamma =C_{p}/C_{v}$ is the ratio of specific heats. The gas pressure *P* follows the ideal gas law, $P=2n\kappa_{B}T$, where electrons and ions have the same number density of *n*, $\kappa_{B}$ is the Boltzmann constant and *T* is the temperature.

Then, the MHD equations are normalized, by choosing the normalization factors $L_{0}$ for length, $\rho_{0}$ for mass density, $B_{0}$ for magnetic field, $u_{0}=B_{0}/\sqrt{\mu_{0}\rho_{0}}$ for velocity, $t_{0}=L_{0}/u_{0}$ for time, $P_{0}=\rho_{0}u_{0}^{2}=B_{0}^{2}/\mu_{0}$ for pressure and $e_{0}=P_{0}$ for total energy density, as [11]

(1) $$\begin{array}{c} \;\frac{\partial \rho^{\prime }}{\partial t^{\prime }}+\nabla^{\prime }\cdot \left(\rho^{\prime }{\bar{u}}^{\prime }\right)=0 \end{array}$$

(2) $$\begin{array}{c} \;\frac{\partial (\rho^{\prime }{\bar{u}}^{\prime })}{\partial t^{\prime }}+\nabla^{\prime }\cdot \left(\rho^{\prime }{\bar{u}}^{\prime }{\bar{u}}^{\prime }+P_{t}^{\prime }\bar{\bar{I}}-{\bar{B}}^{\prime }{\bar{B}}^{\prime }\right)=0 \end{array}$$

(3) $$\begin{array}{c} \;\frac{\partial {\bar{B}}^{\prime }}{\partial t^{\prime }}-\nabla^{\prime }\times \left({\bar{u}}^{\prime }\times {\bar{B}}^{\prime }\right)=0 \end{array}$$

(4) $$\begin{array}{c} \;\frac{\partial e^{\prime }}{\partial t^{\prime }}+\nabla^{\prime }\cdot \left[\left(e^{\prime }+P_{t}^{\prime }\right){\bar{u}}^{\prime }-{\bar{B}}^{\prime }{\bar{B}}^{\prime }\cdot {\bar{u}}^{\prime }\right]=0 \end{array}$$

(5) $$\begin{array}{c} \;\nabla^{\prime }\cdot {\bar{B}}^{\prime }=0 \end{array}$$

where $\alpha^{\prime }=\alpha /\alpha_{0}$, with α, $\alpha_{0}$ and $\alpha^{\prime }$ being the original variable, the normalization factor and the normalized variable, respectively.

The normalized MHD Equations (1)–(5) can be solved by applying the conservative finite-difference scheme that strictly conserves mass, momentum, energy and magnetic flux [12]. However, in low β regions, with $\beta =2\mu_{0}P/B^{2}$, the pressure is computed as the difference between two large numbers, which is prone to numerical errors and usually turns out to be negative [12]. Hence, the normalized MHD equations in semi-conservative form are adopted [12,13]

(6) $$\begin{array}{c} \;\frac{\partial \rho^{\prime }}{\partial t^{\prime }}+\nabla^{\prime }\cdot \left(\rho^{\prime }{\bar{u}}^{\prime }\right)=0 \end{array}$$

(7) $$\begin{array}{c} \;\frac{\partial (\rho^{\prime }{\bar{u}}^{\prime })}{\partial t^{\prime }}+\nabla^{\prime }\cdot \left(\rho^{\prime }{\bar{u}}^{\prime }{\bar{u}}^{\prime }+P^{\prime }\bar{\bar{I}}\right)={\bar{J}}^{\prime }\times {\bar{B}}^{\prime } \end{array}$$

(8) $$\begin{array}{c} \;\frac{\partial {\bar{B}}^{\prime }}{\partial t^{\prime }}=-\nabla^{\prime }\times {\bar{E}}^{\prime } \end{array}$$

(9) $$\begin{array}{c} \;\frac{\partial e^{\prime }}{\partial t^{\prime }}+\nabla^{\prime }\cdot \left[\left(e^{\prime }+P^{\prime }\right){\bar{u}}^{\prime }\right]={\bar{J}}^{\prime }\cdot {\bar{E}}^{\prime } \end{array}$$

(10) $$\begin{array}{c} \;\nabla^{\prime }\cdot {\bar{B}}^{\prime }=0 \end{array}$$

where ${\bar{J}}^{\prime }=\nabla \times {\bar{B}}^{\prime }$, ${\bar{E}}^{\prime }=-{\bar{u}}^{\prime }\times {\bar{B}}^{\prime }$ and $e^{\prime }=P^{\prime }/\left(\gamma -1\right)+\rho^{\prime }u^{\prime 2}/2$.

The MHD Equations (6)–(9) can be reorganized into a conservative form as

$$\begin{array}{c} \;\frac{\partial {\bar{U}}^{\prime }}{\partial t^{\prime }}+\nabla^{\prime }\cdot {\bar{\bar{F}}}^{\prime }={\bar{S}}^{\prime } \end{array}$$

where ${\bar{U}}^{\prime }$ contains all the normalized conservative variables, ${\bar{S}}^{\prime }$ is the source vector and ${\bar{\bar{F}}}^{\prime }$ is the flux tensor, which can be solved as [14]

$$\begin{array}{c} \;{\bar{\bar{F}}}^{\prime }\left({\bar{U}}_{L}^{\prime },{\bar{U}}_{R}^{\prime }\right)=\frac{1}{2}\left({\bar{\bar{F}}}_{L}^{\prime }+{\bar{\bar{F}}}_{R}^{\prime }\right)-\frac{1}{2}\lambda^{\prime }\left({\bar{U}}_{R}^{\prime }-{\bar{U}}_{L}^{\prime }\right) \end{array}$$

where the subscripts *L* and *R* denote the left and right state, respectively, and $\lambda^{\prime }$ is the fastest normalized wave speed. The constrained transport (CT) technique is applied to enforce the divergence-free constraint on the magnetic field in Equation (10) [15]. Structured grid in Cartesian coordinate is adopted in each simulation case. The number of grid points depends on the size of the computational domain, and mesh refinement technique is applied to ensure convergent results.

## 3. Simulation on Earth’s Magnetosphere

In the simulation on the Earth’s magnetosphere, the computational domain is set to $-30\leq x/R_{e}\leq 30$ and $-25\leq y/R_{e},z/R_{e}\leq 25$, with the Earth at the origin, where $R_{e}=6371$ km is the mean Earth radius. A spherical inner boundary is centered at the Earth, with radius of $3R_{e}$. The $\hat{x}$ direction points from the Earth center towards the Sun, the $\hat{z}$ direction is normal to the orbital plane of Earth, and $\hat{y}=\hat{z}\times \hat{x}$. The tilt angle between the Earth rotational axis and $\hat{z}$ is about 23.5°, and the dipole axis of the geomagnetic field is about 11.5° off the rotational axis, leading to a maximum tilt angle about 35° between the dipole axis and $\hat{z}$.

On the inflow boundary of the computational domain, $x=30R_{e}$, the relevant parameters are chosen as n=5 cm${}^{-3}$, $u_{x}=-400$ km/s, $T=2\times 10^{5}$ K and $B_{z}=5$ nT [6]. The pressure is determined from the temperature by the ideal gas law. The normalization factors are $L_{0}=R_{e}$, $n_{0}=1\times 10^{4}$ cm${}^{-3}$, $B_{0}=3.12\times 10^{-5}$ T, $P_{0}=B_{0}^{2}/\mu_{0}=7.74\times 10^{-4}$ Pa, $u_{0}=\sqrt{P_{0}/\rho_{0}}=6807$ km/s and $t_{0}=L_{0}/u_{0}=0.936$ s. The initial number density is n=28 cm${}^{-3}$ on the inner boundary and decreases outwards as a function of $1/r^{3}$ [4]. The temperature in the ionosphere is set to T=1510 K [16], the associated pressure is determined by the ideal gas law and decreases outwards as a function of $1/r^{2}$ [4]. The initial velocity distribution is zero in the whole computational domain.

The magnetosphere-ionosphere coupling (MI coupling) is implemented on the inner boundary in five steps [17,18].

Step 1: Compute the field-aligned current (FAC) density on the inner boundary as

(11) $$\begin{array}{c} J_{\|}=\frac{1}{\mu_{0}}\left[\nabla \times \bar{B}\right]\cdot \hat{b} \end{array}$$

where $\bar{B}={\bar{B}}_{e}+{\bar{B}}_{1}$ is the total magnetic field with direction $\hat{b}$, ${\bar{B}}_{e}$ is the geomagnetic field, and ${\bar{B}}_{1}$ is the perturbation of magnetic field.

Step 2: The FAC density in Equation (11) flows along the geomagnetic field line to the ionosphere, with its magnitude amplified by a factor of $B_{e}^{\mathrm{iono}}/B_{e}^{\mathrm{ib}}$ [17], where $B_{e}^{\mathrm{ib}}$ and $B_{e}^{\mathrm{iono}}$ are the geomagnetic-field strengths on the inner boundary and on the ionosphere, with effective radius of $R_{i}=1.017R_{e}$, respectively. The FAC density mapped from the inner boundary to the ionosphere at $r=R_{i}$ is first interpolated to a grid with resolution of 1° in both latitude and longitude. Then, a spherical-harmonics expansion is applied to the interpolated data to derive the FAC density on the ionosphere.

Step 3: The ionospheric potential, Ψ, satisfies [17]

(12) $$\begin{array}{c} J_{\|}\left(r=R_{i}\right)\cos\delta =\nabla \cdot \left(\bar{\bar{\Sigma }}\cdot \nabla \Psi \right) \end{array}$$

where

$$\begin{array}{c} \bar{\bar{\Sigma }}=\left[\begin{array}{cc} \sigma_{p}/\cos^{2}\delta & -\sigma_{h}/\cos\delta \\ \sigma_{h}/\cos\delta & \sigma_{p} \end{array}\right] \end{array}$$

is the ionospheric conductance tensor, $\sigma_{p}$ and $\sigma_{h}$ are Pedersen conductance and Hall conductance, respectively, δ is the dip angle of geomagnetic field, which is related to the colatitude θ as $\cos\delta =-2\cos\theta /\sqrt{1+3\cos^{2}\theta }$. In the dayside ionosphere, electron precipitation and ionization by solar radiation determine Pedersen and Hall conductances [17]. Empirical formulas of ionospheric conductances in terms of the solar zenith angle ξ are available [19]. In this work, we set $\sigma_{p}=5$ S and $\sigma_{h}=0$ [6]. A generalized minimum residual (GMRES) method [20] is applied to solve Equation (12) for the ionospheric potential.

Step 4: The potential $\Psi^{\mathrm{ib}}$ at a point on the inner boundary is set equal to its counterpart at $r=R_{i}$, along the geomagnetic field line. The potentials at the grid points on the ionosphere are mapped onto the inner boundary, which are then interpolated to a grid with resolution of 1° in both latitude and longitude. Next, another spherical-harmonics expansion is applied to the interpolated data to derive the potential distribution on the inner boundary.

Step 5: The electric field on the inner boundary is computed as

$$\begin{array}{c} {\bar{E}}^{\mathrm{ib}}=-\nabla \Psi^{\mathrm{ib}} \end{array}$$

and the tangential velocity of plasma is estimated as

$$\begin{array}{c} \bar{v}=\frac{{\bar{E}}^{\mathrm{ib}}\times {\bar{B}}^{\mathrm{ib}}}{{\left|{\bar{B}}^{\mathrm{ib}}\right|}^{2}} \end{array}$$

where ${\bar{B}}^{\mathrm{ib}}={\bar{B}}_{e}^{\mathrm{ib}}+{\bar{B}}_{1}^{\mathrm{ib}}$. The radial velocity on the inner boundary is neglected. The tangential velocity at a nearby grid point in the computational domain is bilinearly interpolated from four neighboring points surrounding the projection of the grid point on the inner boundary along the geomagnetic field line.

**Figure 1:**
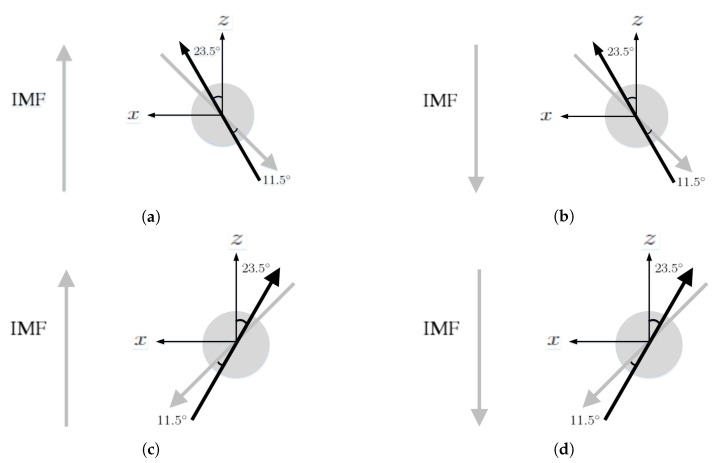
Scenarios of solar-wind blowing on Earth. (**a**) northward interplanetary magnetic field (IMF) in June, (**b**) southward IMF in June, (**c**) northward IMF in December, (**d**) southward IMF in December. The rotational axis (black arrow) tilts by 23.5° from the orbital normal and the dipole axis (grey arrow) tilts by 11.5° from the rotational axis.

Figure 1 shows four different scenarios in the simulation of solar-wind blowing on the Earth’s magnetosphere. The field strength in the northward interplanetary magnetic field (IMF) is $B_{z}=5$ nT and that in the southward IMF is $B_{z}=-5$ nT.

**Figure 2:**
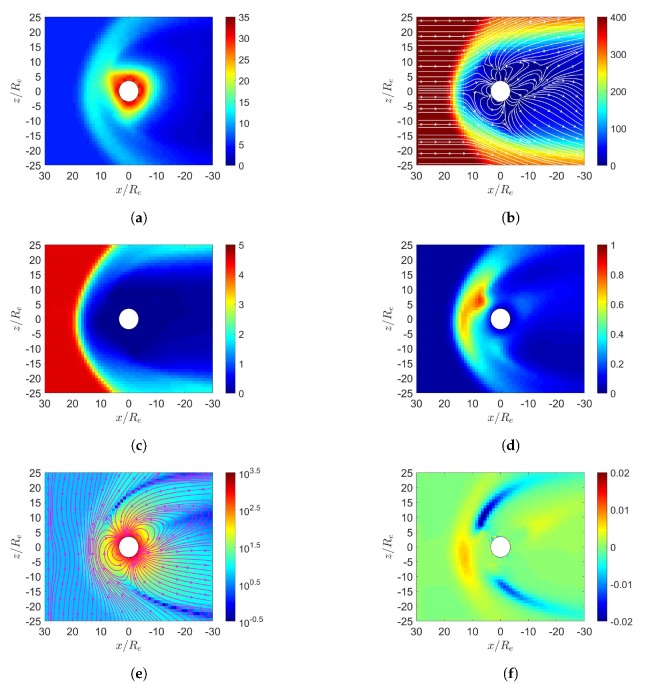
Distributions around Earth of magnetosphere. (**a**) *n* (cm${}^{-3}$), (**b**) ${\bar{u}}_{xz}$ (km/s), (**c**) magnetosonic Mach number, (**d**) *P* (nPa), (**e**) ${\bar{B}}_{xz}$ (nT), (**f**) $J_{y}$ (μ/m${}^{2}$); northward IMF in June, t=27 min.

Figure 2 shows the simulation results under northward IMF in June. The solar wind reaches the Earth’s magnetosphere, induces a bow shock in front of the magnetopause with nose cone at $x≃16R_{e}$, raises the temperature, decelerates and detours around the magnetopause. Figure 2a shows the number density increases behind the bow shock, Figure 2d shows the pressure increases in the same region, with the highest pressure in the dayside magnetosphere shifted towards the north magnetic pole. Figure 2b shows that the flow pattern of ${\bar{u}}_{xz}$ becomes complicated around the inner boundary, partly attributed to the reconnection or convecting electric-field [6]. Strong velocity shear is observed in the magnetopause where Kelvin–Helmholtz instability (KHI) can potentially be triggered and transfers some solar-wind energy to the magnetosphere [4]. The KHI is not observed because finer resolution and numerical perturbation are needed.

There are two possible mechanisms to account for sunward flows in the dayside magnetosphere. If reconnection takes place near the dayside magnetosphere, particles will flow in to replenish the depleted region. From a reference frame fixed to the Earth, convecting electric field is induced by the solar wind as ${\bar{E}}_{\mathrm{sw}}=-{\bar{u}}_{\mathrm{sw}}\times {\bar{B}}_{\mathrm{sw}}$, which drives an $\bar{E}\times \bar{B}$ drift in the sunward direction.

Figure 2c shows the magnetosonic Mach number, $M_{\mathrm{ms}}=u/u_{\mathrm{ms}}$ [21], where $u_{\mathrm{ms}}=\sqrt{c_{s}^{2}+u_{A}^{2}}$, $c_{s}=\sqrt{\gamma P/\rho }$ and $u_{A}=B/\sqrt{\mu_{0}\rho_{0}}$. The dynamic ram pressure, $\rho u^{2}$, of solar wind and the magnetospheric magnetic pressure, $B^{2}/\left(2\mu_{0}\right)$, are balanced at the standoff distance of magnetopause [2]

$$\begin{array}{c} R_{\mathrm{mp}}={\left(\frac{a^{2}B_{e}^{2}}{2\mu_{0}m_{p}n_{\mathrm{sw}}u_{\mathrm{sw}}^{2}}\right)}^{1/6}R_{e} \end{array}$$

which is about $14.7R_{e}$ if a=2.4, $B_{e}=3\times 10^{-5}$ T, $n_{\mathrm{sw}}=5$ cm${}^{-3}$ and $u_{\mathrm{sw}}=400$ km/s.

Figure 2e shows that the reconnection region in the northern dayside is pulled towards the sun, while that in the southern dayside is pushed away from the sun, due to the dipole-axis orientation in June. The northward IMF is parallel to the geomagnetic field in the dayside magnetosphere, the incident solar wind tends to compress the geomagnetic field lines to where the geomagnetic field is antiparallel to the northward IMF, leading to cusp reconnection. A cusp reconnection region is also observed in the nightside [6]. Figure 2f shows that strong current density $J_{y}$ is induced around the bow shock, generating Lorentz force, $\bar{J}\times \bar{B}$, which points in the *x* direction and decelerates the solar wind.

**Figure 3:**
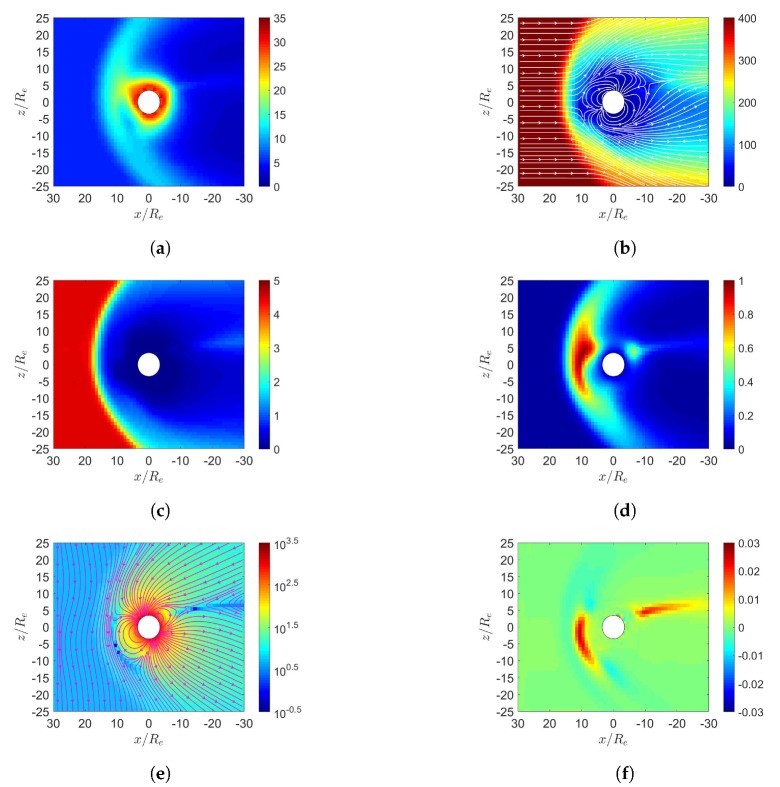
Distributions around magnetosphere of Earth, (**a**) *n* (cm${}^{-3}$), (**b**) ${\bar{u}}_{xz}$ (km/s), (**c**) magnetosonic Mach number, (**d**) *P* (nPa), (**e**) ${\bar{B}}_{xz}$ (nT), (**f**) $J_{y}$ (μ/m${}^{2}$); southward IMF in June, t=27 min.

Figure 3 shows the simulation results under southward IMF in June. In Figure 3e, magnetic reconnection occurs in the dayside magnetosphere where the southward IMF is antiparallel to the geomagnetic field. The solar wind further pushes leeward both the north and south tail lobes [7]. When these two lobes contact each other, reconnection takes place and releases the magnetic energy to cause magnetospheric substorm [7]. A completely detached plasmoid is also observed in the magnetotail. Figure 3d shows that the highest pressure in the dayside magnetosphere is shifted towards the north magnetic pole, stronger than its counterpart in Figure 2d.

**Figure 4:**
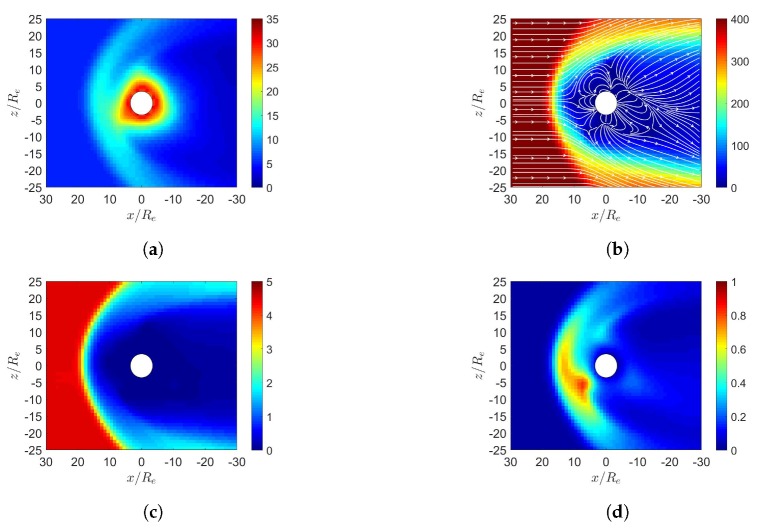

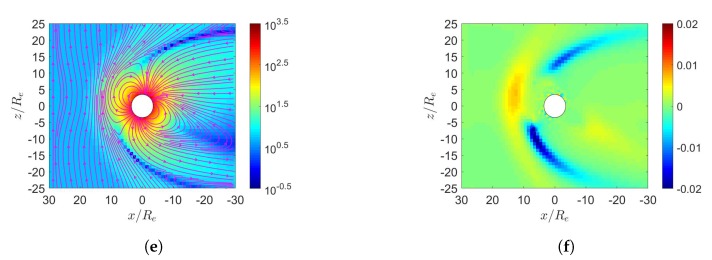
Distributions around magnetosphere of Earth. (**a**) *n* (cm${}^{-3}$), (**b**) ${\bar{u}}_{xz}$ (km/s), (**c**) magnetosonic Mach number, (**d**) *P* (nPa), (**e**) ${\bar{B}}_{xz}$ (nT), (**f**) $J_{y}$ (μ/m${}^{2}$); northward IMF in December, t=27 min.

Figure 4 shows the simulation results under northward IMF in December. Figure 4d shows that the highest pressure in the dayside magnetosphere is shifted towards the south magnetic pole. Figure 4e shows the northern cusp reconnection region is pushed away from the sun while the southern cusp is pulled towards the sun, due to the dipole-axis orientation in December.

**Figure 5:**
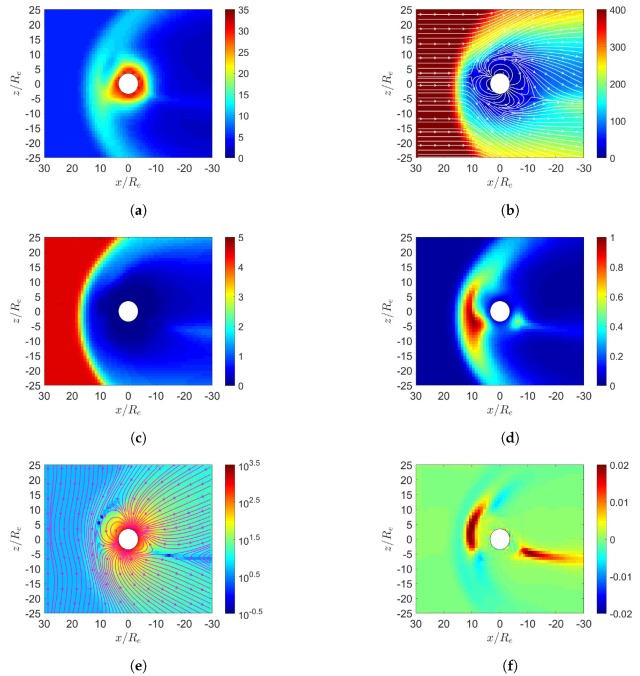
Distributions around Earth of (**a**) *n* (cm${}^{-3}$), (**b**) ${\bar{u}}_{xz}$ (km/s), (**c**) magnetosonic Mach number, (**d**) *P* (nPa), (**e**) ${\bar{B}}_{xz}$ (nT), (**f**) $J_{y}$ (μ/m${}^{2}$); southward IMF in December, t=27 min.

Figure 5 shows the simulation results under southward IMF in December. Figure 5d shows the highest pressure in the dayside magnetosphere is shifted towards the south magnetic pole, with higher pressure than its counterpart in Figure 4d. It is observed that the plasmoid in Figure 5e lies below the orbital plane and that in Figure 3e lies above the orbital plane, due to different orientations of dipole axis.

## 4. Simulation on Mercury’s Magnetosphere

In the simulation on the Mercury’s magnetosphere, the computational domain is set to $-6\leq x/R_{M},y/R_{M},z/R_{M}\leq 6$, with the Mercury located at the origin, where $R_{M}$ is the radius of Mercury. The $\hat{x}$ direction points from the Mercury center towards the Sun, the $\hat{z}$ direction is normal to the orbital plane of Mercury. The initial number density and pressure in the whole computational domain are set to n=10 cm${}^{-3}$, u=0 km/s and P=0.19 nPa [22]. The magnetic field of Mercury is represented as [22]

(13) $$\begin{array}{c} \;B_{x}=-\frac{\mu_{0}M_{H}}{4\pi r^{5}}3xz \end{array}$$

(14) $$\begin{array}{c} \;B_{y}=-\frac{\mu_{0}M_{H}}{4\pi r^{5}}3yz \end{array}$$

(15) $$\begin{array}{c} \;B_{z}=-\frac{\mu_{0}M_{H}}{4\pi r^{5}}3\left(z^{2}-r^{2}\right) \end{array}$$

where $M_{H}=4.36\times 10^{19}$ A · m${}^{2}$ is the dipole moment of Mercury, with the magnetic field of 300 nT at the equator of Mercury [22]. The tilt angles of both the rotational axis and the dipole axis are 0° [3]. The parameters of the inflow solar wind, specified at $x=6R_{M}$, are n=35 cm${}^{-3}$, $u_{x}=-400$ km/s, P=0.19 nPa and $B_{z}=5$ nT [22]. The normalization factors are $L_{0}=R_{M}=2440$ km, $n_{0}=10$ cm${}^{-3}$, $B_{0}=300$ nT, $P_{0}=B_{0}^{2}/\mu_{0}=71.6$ nPa, $u_{0}=\sqrt{P_{0}/\rho_{0}}=2070$ km/s and $t_{0}=L_{0}/u_{0}=1.18$ s. The magnetic field of Mercury is about 1 % that of the Earth, thus a smaller spherical inner boundary of radius $1R_{M}$ is chosen. The effective conductance in the Mercury’s ionosphere is not well known, hence relevant parameters are estimated from the initial values of the Mercury’s ionosphere [22].

**Figure 6:**
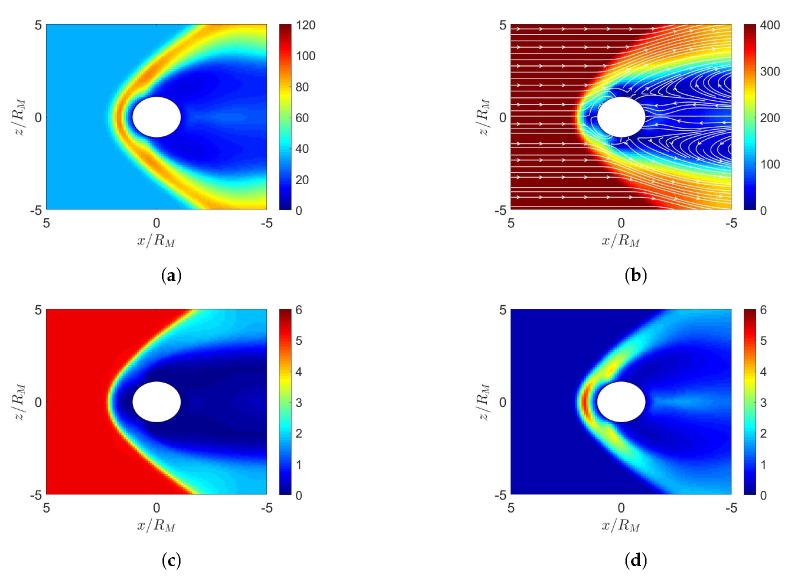

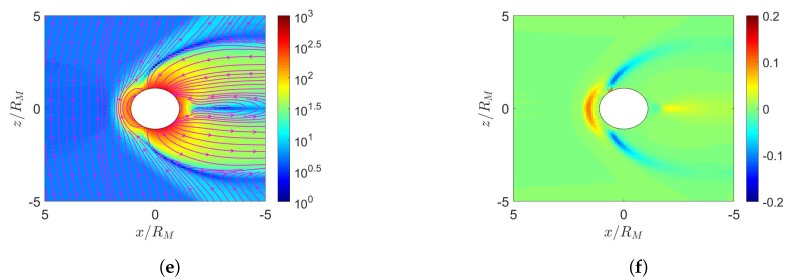
Distributions around Mercury of (**a**) *n* (cm${}^{-3}$), (**b**) ${\bar{u}}_{xz}$ (km/s), (**c**) magnetosonic Mach number, (**d**) *P* (nPa), (**e**) ${\bar{B}}_{xz}$ (nT), (**f**) $J_{y}$ (μA/m${}^{2}$); northward IMF, t= 1 min 58 s.

Figure 6 shows the simulation results under northward IMF. The number density in Figure 6a and the pressure in Figure 6d indicate that when the solar wind reaches the Mercury’s magnetosphere, a bow shock is induced in front of the magnetopause, with nose cone at $x≃2R_{M}$. The bow shock appears considerably closer to the Mercury surface than its counterpart to the Earth surface, as shown in Figure 2 and Figure 4. Figure 6e shows that the magnetic field in the cusp reconnection region is very weak. Figure 6f shows strong current density induced near the bow shock, generating Lorentz force, $\bar{J}\times \bar{B}$, which points in the *x* direction and decelerates the solar wind.

**Figure 7:**
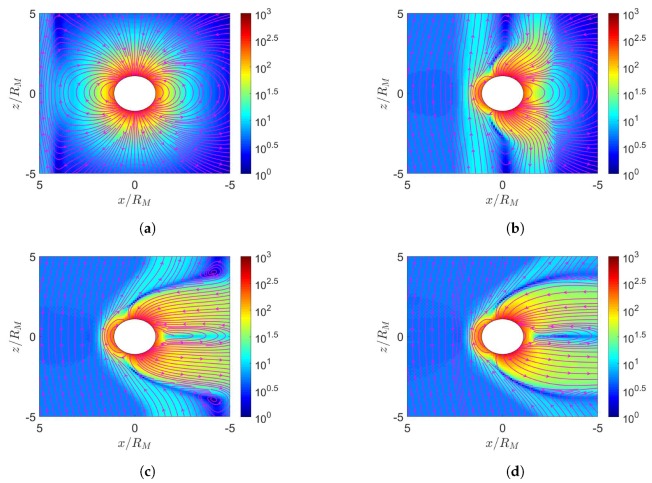
Time evolution of Mercury’s magnetic-field distribution under northward IMF. (**a**) t=12.8 s, (**b**) t=51.5 s, (**c**) t=90.0 s, (**d**) t=128.8 s.

Figure 7 shows the time evolution of Mercury’s magnetic-field distribution under northward IMF. The magnetic-field lines in the dayside magnetosphere are compressed leeward by the solar wind. Two cusp reconnection regions are also pushed and bent towards the nightside. The magnetic-field lines in the nightside magnetosphere are compressed towards the orbital plane, forming another reconnection region in the magnetotail.

**Figure 8:**
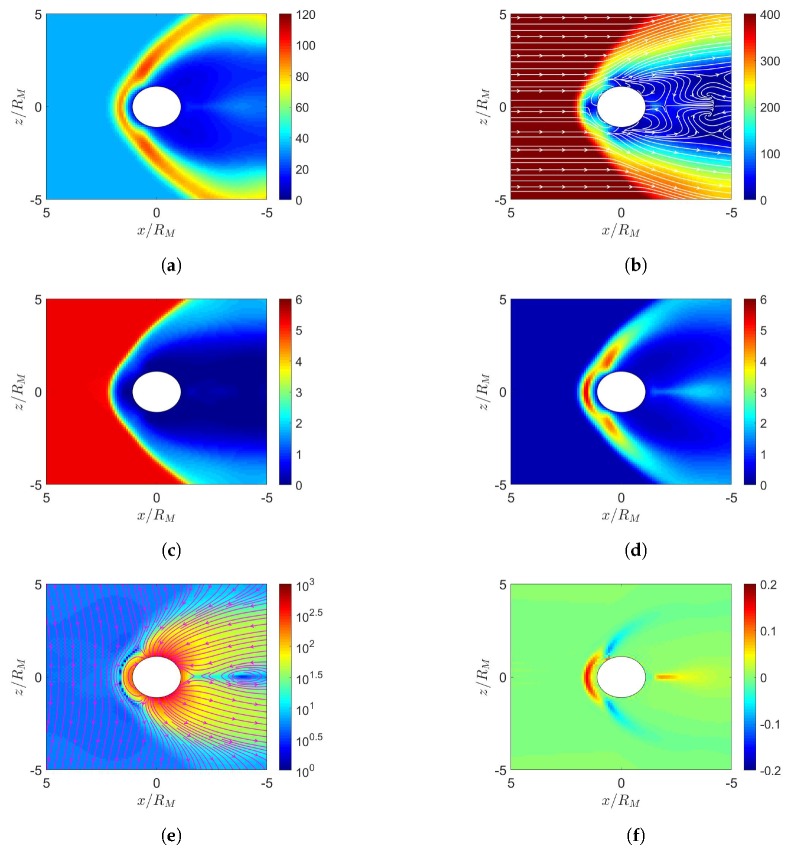
Distributions around Mercury of (**a**) *n* (cm${}^{-3}$), (**b**) ${\bar{u}}_{xz}$ (km/s), (**c**) magnetosonic Mach number, (**d**) *P* (nPa), (**e**) ${\bar{B}}_{xz}$ (nT), (**f**) $J_{y}$ (μ/m${}^{2}$); southward IMF, t= 1 min 58 s.

Figure 8 shows the simulation results under southward IMF. Figure 8e shows magnetic reconnection in the dayside magnetosphere, in the vicinity of (−2,0). Figure 8b shows that plasma is pushed towards the Mercury and detours around it. The induced currents in both Figure 6f and Figure 8f are on the same order-of-magnitude as their counterparts in the Earth’s magnetosphere. The solar wind near the reconnection region flows tailwards, pushing the two lobes around the magnetotail to squeeze a current sheet in between. As the magnetic-field lines in these two lobes contact each other, another reconnection takes place and magnetic energy is released. Figure 8e shows a plasmoid in the magnetotail. Compared with Figure 3e on the Earth’s magnetosphere, a plasmoid appears in $x<-30R_{e}$, not completely displayed, which is farther away from the planet itself because the geomagnetic field is relatively stronger than that of Mercury.

**Figure 9:**
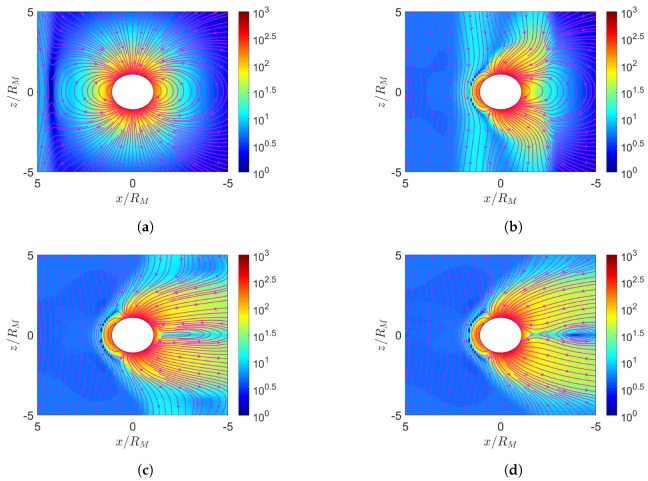
Time evolution of Mercury’s magnetic field distribution under southward IMF. (**a**) t=12.8 s, (**b**) t=51.5 s, (**c**) t=90 s, (**d**) t=128.8 s.

Figure 9 shows the time evolution of Mercury’s magnetic field distribution under southward IMF. Magnetic reconnection occurs in the dayside magnetosphere, creating open fluxes in the magnetotail [7]. The accumulated energy in the magnetotail is released where the two lobes meet, forming a detached plasmoid in the magnetotail, as shown in Figure 9d.

## 5. Simulation on Jupiter’s Magnetosphere

The volcanic activities in Io, which lies at a radial distance of 5.9 $R_{J}$ from Jupiter, ejects about metric ton of mass per second into the Jovian magnetosphere [23]. The neutral matter from Io accumulates primarily along its orbit and is eventually ionized to form a torus. Similar effect was observed in Saturn’s ionosphere, with rings, icy satellites and Titan providing neutral gas sources [24]. Both Saturn and Jupiter rotate fast, with their centrifugal force throwing protons outwards [25]. The magnetic field in the Jovian magnetosphere is modified by the outward moving plasma, generating Lorentz force, $\bar{J}\times \bar{B}$, to drag the plasma towards corotation [23]. From another point of view, the elastic collisions between neutral particles in Jupiter’s atmosphere and the plasma in Jupiter’s ionosphere provide momentum for the latter to drag the magnetospheric plasma within $15R_{J}$ to near corotation with Jupiter [23]. However, at radial distance between 15 and 30 $R_{J}$, the dragging force is weaker and can sustain only 75 % of corotation [26]. The outward-moving heavy plasma caused by fast Jupiter rotation (9.92 hours in period) stretches the magnetic-field distribution and induces reconnection. A plasmoid is formed in the magnetotail [24], carrying away some plasma from the inner magnetosphere [27].

**Figure 10:**
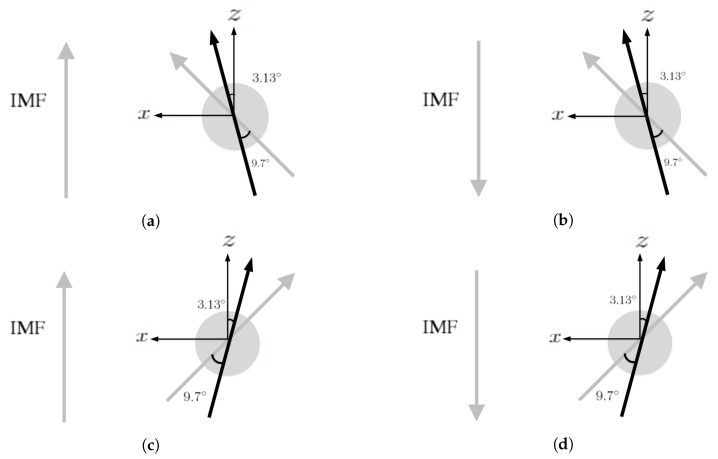
Scenarios of solar-wind blowing on Jupiter. (**a**) initial dipole tilts towards sun, northward IMF, (**b**) initial dipole tilts towards sun, southward IMF, (**c**) initial dipole tilts away from sun, northward IMF, (**d**) initial dipole tilts away from sun, southward IMF. The rotational axis (black arrow) tilts by 3.13° from the orbital normal and the dipole axis (grey arrow) tilts by 9.7° from the rotational axis, and lies on xz plane at t=0.

Figure 10 shows the four scenarios to simulate solar-wind blowing on the Jupiter. The computational domain is set to $-120R_{J}\leq x\leq 120R_{J}$, $-80R_{J}\leq y,z\leq 80R_{J}$, with Jupiter located at the origin, where $R_{J}$ is the radius of Jupiter. Jupiter center points towards the Sun in the $\hat{x}$ direction and $\hat{z}$ is normal to the orbital plane of Jupiter. The grid size is set to $3R_{J}$ in $r>25R_{J}$ and $1.5R_{J}$ in $r<25R_{J}$. A spherical inner boundary is placed at $r=15R_{J}$.

The inflow parameters, at $x=120R_{J}$, are set to n=1 cm${}^{-3}$, $u_{x}=-300$ km/s, T=5000 K and $|B_{z}|=0.78$ nT [23]. The initial conditions in the region of $15R_{J}\leq r\leq 100R_{J}$ are set to n=20/r cm${}^{-3}$, u=0 km/s and P=0.35/r nPa [8]. The parameters on the inner boundary are set to n=20/15 cm${}^{-3}$ and P=0.35/15 nPa [8]. Since the magnetosphere in $r<15R_{J}$ is nearly corotating with Jupiter, the azimuthal velocity on the inner boundary is set to $u_{\phi }=15\Omega R_{J}$ km/s, where Ω (rad/s) is the Jovian angular velocity. In the region of $r\geq 100R_{J}$, the solar wind is approximated as uniform [8]. The magnetic field of Jupiter is approximated as a dipole field, with the magnetic field of 4.28 Gauss at the equator [28]. The normalization factors are $L_{0}=R_{J}=71,398$ km, $n_{0}=1\times 10^{6}$ cm${}^{-3}$, $B_{0}=4.2\times 10^{-4}$ T, $P_{0}=B_{0}^{2}/\mu_{0}=0.14$ Pa, $u_{0}=\sqrt{P_{0}/\rho_{0}}=9200$ km/s and $t_{0}=L_{0}/u_{0}=7.76$ s.

The parameters, $\rho ,\bar{u},\bar{B}$ and *P*, are fixed on the inner boundary. The mass loading from Io is not explicitly included in the MHD equations. The net transport of ions, estimated as $\int \;\;\;\;\int \;\;\;\;\;\;\;\;◯n\bar{u}\cdot d\bar{a}$ on a sphere of radius $r=21R_{J}$, in the four scenarios shown in Figure 10a–d is computed as $3.28\times 10^{27}$, $3.7\times 10^{27}$, $3\times 10^{27}$ and $3.4\times 10^{27}$ ions/s, respectively. Note that the estimated value is $3\times 10^{28}$ ions/s in [29].

**Figure 11:**
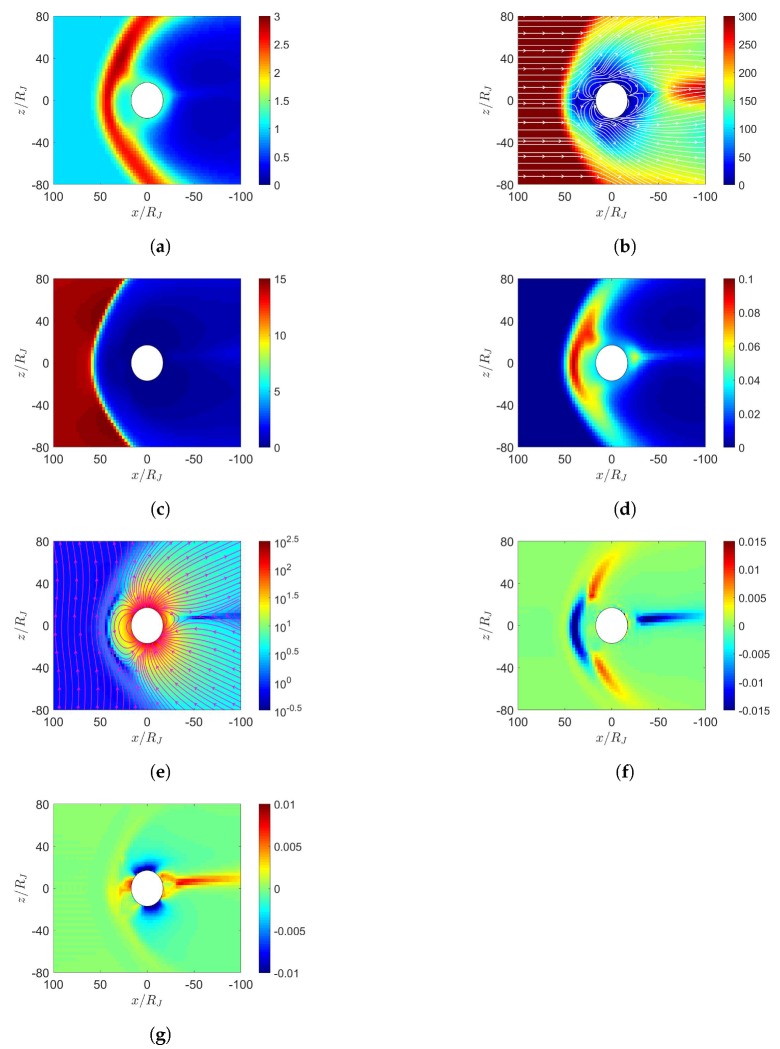
Distributions around Jupiter of (**a**) *n* (cm${}^{-3}$), (**b**) ${\bar{u}}_{xz}$ (km/s), (**c**) magnetosonic Mach number, (**d**) *P* (nPa), (**e**) ${\bar{B}}_{xz}$ (nT), (**f**) $J_{y}$ (nA/m${}^{2}$), (**g**) $J_{r}$ (nA/m${}^{2}$); maximum dipole tilt points towards sun at t=0, under northward IMF, t=21.19 h.

Figure 11 shows the simulation results with maximum dipole tilt pointing towards sun at t=0, under northward IMF. When the solar wind reaches the Jovian magnetosphere, a bow shock is induced in front of the magnetopause, with nose cone at $x≃65R_{J}$, comparable to the observed position between 50 and 100 $R_{J}$ [30]. Around the nose cone, the number density increases as in Figure 11a and the pressure also increases as in Figure 11d. Note that all ions are assumed to be protons in this work. During the Cassini excursion into the Jovian magnetosphere on 10 January 2001, observation showed that the Jovian magnetosphere is dominated by S (25 % S${}^{+}$, 42 % S${}^{2+}$ and 33 % S${}^{3+}$) and O (96 % O${}^{+}$ and 4% O${}^{2+}$) [31]. The centrifugal force and plasma pressure carried by protons are weaker than those by S and O, leading to closer bow shock in the simulation than that by observation [8,23]. By the same argument, including the mass loading from Io is expected to push the bow shock farther away [23].

Figure 11e shows that the solar wind induces a dayside reconnection region and pushes the magnetic fluxes towards both the northern and the southern tail lobes. As the two lobes contact each other, Dungey-cycle reconnection takes place and a fully detached plasmoid emerges [7]. The topology of magnetic field distribution appears similar to that of the Earth’s magnetosphere under southward IMF. Note that the Jovian magnetic dipole is in opposite polarity to that of the Earth. Figure 11f shows the induced current density $J_{y}$, which flows in the −y direction near the magnetopause, generating a Lorentz force, $\bar{J}\times \bar{B}$, to decelerate the solar wind.

Figure 11g shows the corotation-enforcing current $J_{r}$, which concentrates near the orbital plane around $x=-30∼-100R_{J}$, generating a Lorentz force, $\bar{J}\times \bar{B}$, in the ϕ direction (corotation direction) to drag the plasma around Jupiter. Note that both $J_{y}$ and $J_{r}$ are on the order of nA/m${}^{2}$, substantially smaller than their counterparts around Earth and Mercury, which are on the order of μA/m${}^{2}$, because the solar wind is relatively weaker around Jupiter.

**Figure 12:**
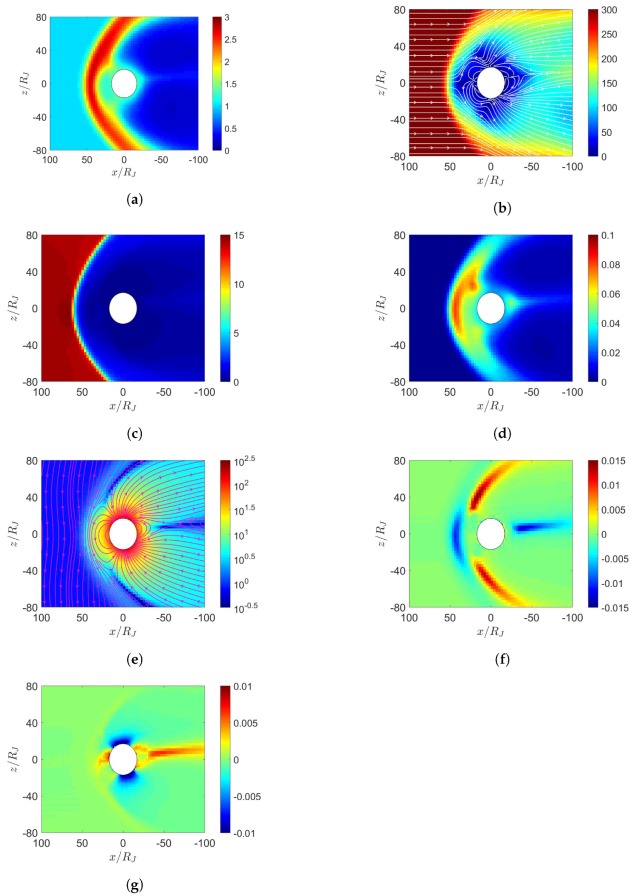
Distributions around Jupiter of (**a**) *n* (cm${}^{-3}$), (**b**) ${\bar{u}}_{xz}$ (km/s), (**c**) magnetosonic Mach number, (**d**) *P* (nPa), (**e**) ${\bar{B}}_{xz}$ (nT), (**f**) $J_{y}$ (nA/m${}^{2}$), (**g**) $J_{r}$ (nA/m${}^{2}$); maximum dipole tilt points towards sun at t=0, under southward IMF, t=21.19 h.

Figure 12 shows the simulation results with maximum dipole tilt pointing towards sun at t=0, under southward IMF. Figure 12e shows two reconnection cusp regions where Vasyliunas cycle takes place. The outward-moving plasma stretches the magnetic field lines to induce another reconnection in the magnetotail, forming a detached plasmoid.

The Jovian magnetosphere under northward IMF, as in Figure 11e, shows similar characteristics to the Earth’s magnetosphere under southward IMF, as in Figure 2e. In both scenarios, the IMF is antiparallel to the planetary dipole field in the dayside magnetosphere. Similarly, the Jovian magnetosphere under southward IMF, as in Figure 12e, shows similar characteristics to the Earth’s magnetosphere under northward IMF, as in Figure 3e, in which the IMF is parallel to the planetary dipole field in the dayside magnetosphere.

**Figure 13:**
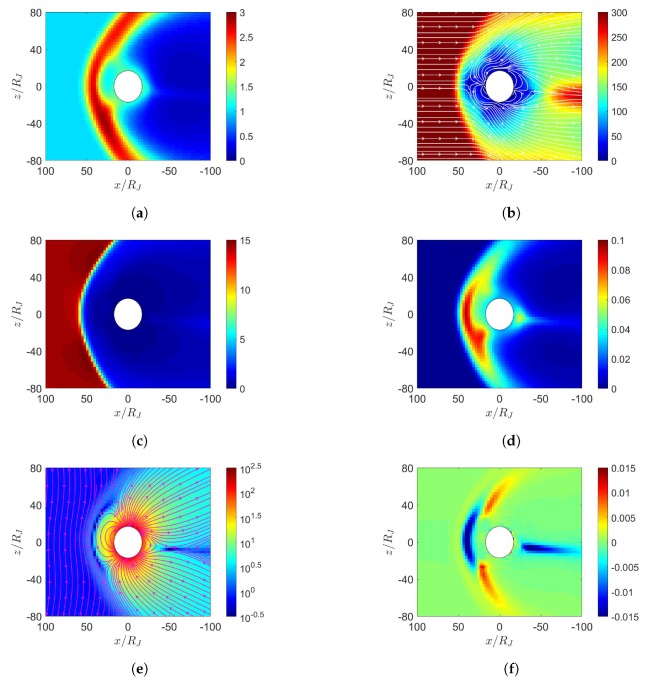

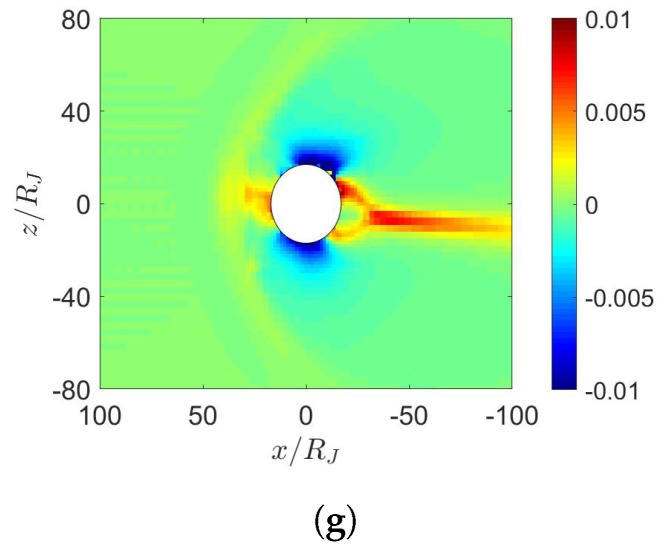
Distributions around Jupiter of (**a**) *n* (cm${}^{-3}$), (**b**) ${\bar{u}}_{xz}$ (km/s), (**c**) magnetosonic Mach number, (**d**) *P* (nPa), (**e**) ${\bar{B}}_{xz}$ (nT), (**f**) $J_{y}$ (nA/m${}^{2}$), (**g**) $J_{r}$ (nA/m${}^{2}$); maximum dipole tilt points away from sun at t=0, under northward IMF, t=21.19 h.

Figure 13 shows the simulation results with maximum dipole tilt pointing away from the sun at t=0, under northward IMF. The highest pressure in the dayside magnetosphere in Figure 11d and Figure 13d appear towards the north and the south magnetic pole, respectively, due to different orientations of dipole tilt. The plasmoid in the magnetotail in Figure 11e and Figure 13e lie above and below the orbital plane, respectively, due to the same reason.

**Figure 14:**
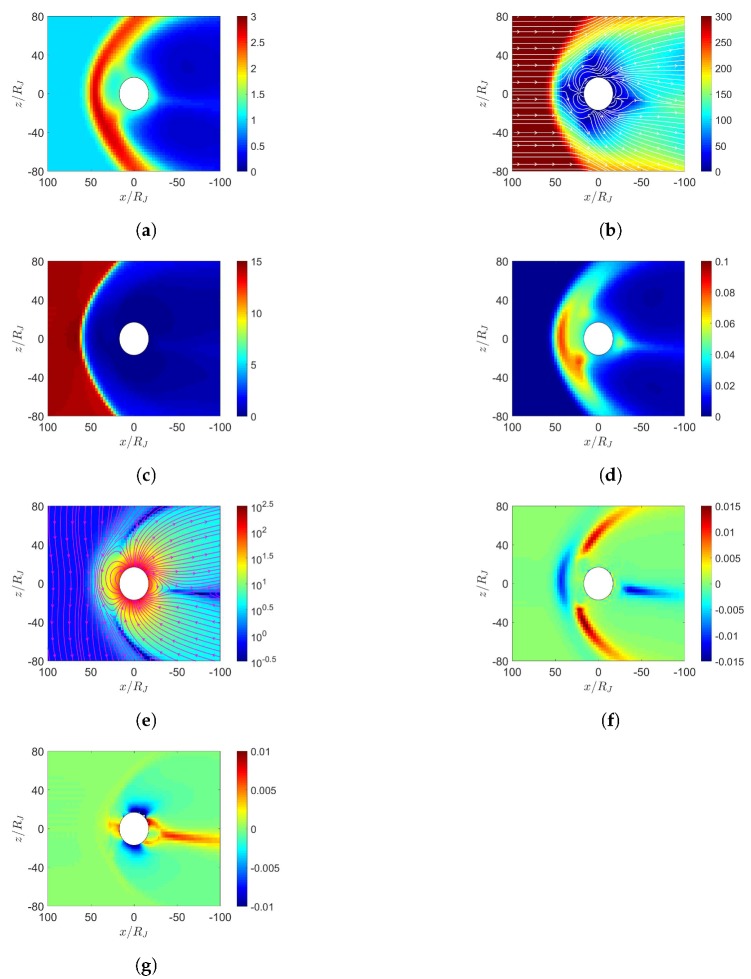
Distributions around Jupiter of (**a**) *n* (cm${}^{-3}$), (**b**) ${\bar{u}}_{xz}$ (km/s), (**c**) magnetosonic Mach number, (**d**) *P* (nPa), (**e**) ${\bar{B}}_{xz}$ (nT), (**f**) $J_{y}$ (nA/m${}^{2}$), (**g**) $J_{r}$ (nA/m${}^{2}$); maximum dipole tilt points away from sun at t=0, under southward IMF, t=21.19 h.

Figure 14 shows the simulation results with maximum dipole tilt pointing away from the sun at t=0, under southward IMF. The highest pressure in the dayside magnetosphere in Figure 12d and Figure 14d appear towards the north and the south magnetic pole, respectively, due to different orientations of dipole tilt. The northern reconnection cusp region is pushed away from the sun, and that the southern cusp is pulled towards the sun.

## 6. Simulation on Uranus’s Magnetosphere

In the simulation on the Uranus’s magnetosphere, the computational domain is set to $-70R_{U}\leq x\leq 70R_{U}$, $-50R_{U}\leq y,z\leq 50R_{U}$, with Uranus at the origin, where $R_{U}$ is the radius of Uranus and the grid size is $2R_{U}$. The inflow parameters, at $x=70R_{U}$, are n=0.1 cm${}^{-3}$, $u_{x}=-450$ km/s, T=54,541 K and $B_{z}=\pm 0.22$ nT [10,32]. The inflow parameters in [10] were based on the Voyager-2 observation data, and the magnetic field of the solar wind was set to zero as the observed magnetic field undulated about zero. In this work, we choose $B_{z}=\pm 0.22$ nT in the solar wind [32]. The inner boundary is placed at $r=5R_{U}$, with n=0.086 cm${}^{-3}$ and $P=1.987\times 10^{-4}$ nPa [10]. The normalization factors are $L_{0}=R_{U}=25,559$ km, $n_{0}=10^{5}$ cm${}^{-3}$, $B_{0}=2.2836\times 10^{-5}$ T, $P_{0}=B_{0}^{2}/\mu_{0}=4.15\times 10^{-4}$ Pa, $u_{0}=\sqrt{P_{0}/\rho_{0}}=1580$ km/s, and $t_{0}=L_{0}/u_{0}=16.18$ s.

The Uranus’s magnetic field is approximated as a dipole field, with the magnitude of 22,836 nT at its equator, and the dipole center is offset from the Uranus center by $0.31R_{U}$ [10]. The rotational axis *R* is tilted by 97.9° from the orbital normal, and the angle between the magnetic dipole axis and the rotational axis is 58.61° [10]. Looking at the Uranus from the sun, the magnetic dipole axis rotates counterclockwise [10]. The magnetosphere of the Uranus switches between an open configuration and a closed one during each Uranus day, which is about 17.24 sidereal hours [9].

**Figure 15:**
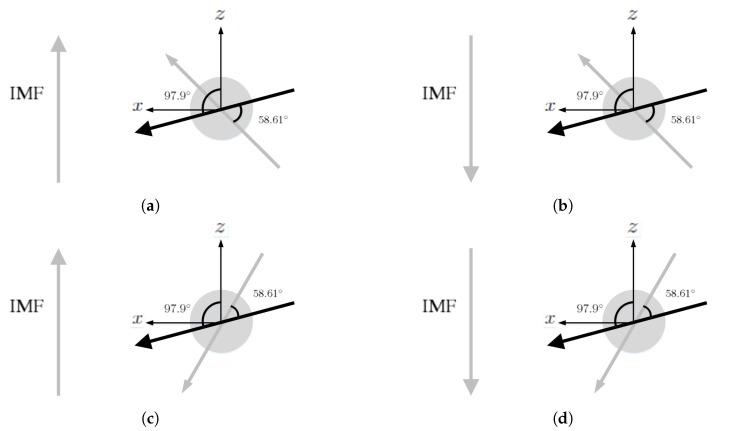
Scenarios of solar-wind blowing on Uranus. (**a**) initial dipole tilts up and towards the sun, northward IMF, (**b**) initial dipole tilts up and towards the sun, southward IMF, (**c**) initial dipole tilts down and towards the sun, northward IMF, (**d**) initial dipole tilts down and towards the sun, southward IMF. The rotational axis (black arrow) is tilted by 97.9° from the orbital normal and the dipole axis (grey arrow) is tilted by 58.61° from the rotational axis.

Figure 15 shows four scenarios of solar wind blowing the Uranus. Within the first hour when solar wind reaches the Uranus magnetosphere, the planetary is simulated as a stationary sphere with a fixed magnetic dipole depicted in Figure 15, and the inertial force and relative rotation of solar wind are neglected in the simulation [10].

**Figure 16:**
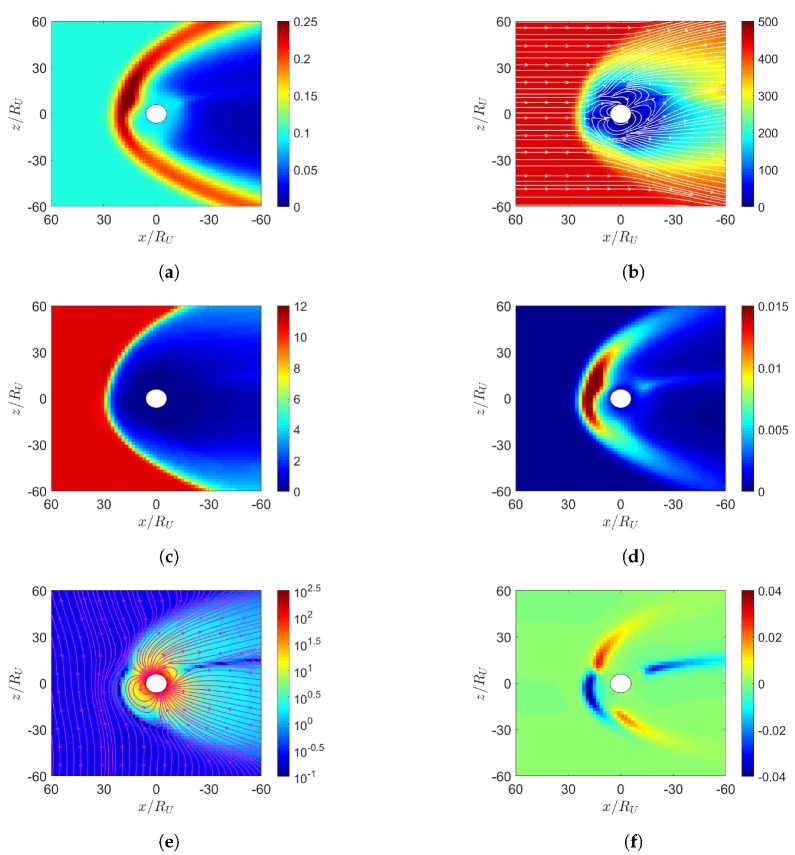
Distributions around Uranus of (**a**) *n* (cm${}^{-3}$), (**b**) ${\bar{u}}_{xz}$ (km/s), (**c**) magnetosonic Mach number, (**d**) *P* (nPa), (**e**) ${\bar{B}}_{xz}$ (nT), (**f**) $J_{y}$ (nA/m${}^{2}$); initial dipole tilts up and towards the sun, northward IMF, t=85.4 min.

Figure 16 shows the simulation results under northward IMF, with the initial dipole tilting up with respect to the orbital plane. When the solar wind reaches the Uranus magnetosphere, a bow shock is induced in front of the magnetopause, with nose cone at $x≃24R_{U}$, where number density increases as in Figure 16a and pressure increases as in Figure 16d. Figure 16e shows magnetic field reconnection in the dayside magnetosphere. The contrast of magnetic-field strength between the dayside reconnection site and the background solar wind outside the bow shock is smaller than 10, less obvious than those in Earth, Mercury and Jupiter. The magnetic field in the dayside magnetosphere is antiparallel to the northward IMF, leading to reconnection and open field configuration.

The structures of bow shock, magnetopause and reconnection site are asymmetric about the orbital plane due to significant dipole tilt. Figure 16f shows that magnetopause current is generated around $x≃18R_{U}$, generating a Lorentz force, $\bar{J}\times \bar{B}$, to impede the solar wind. The induced current is on the order of nA/m${}^{2}$, comparable to that in Jupiter but substantially smaller than those in Earth and Mercury, which are on the order of μA m${}^{-2}$.

**Figure 17:**
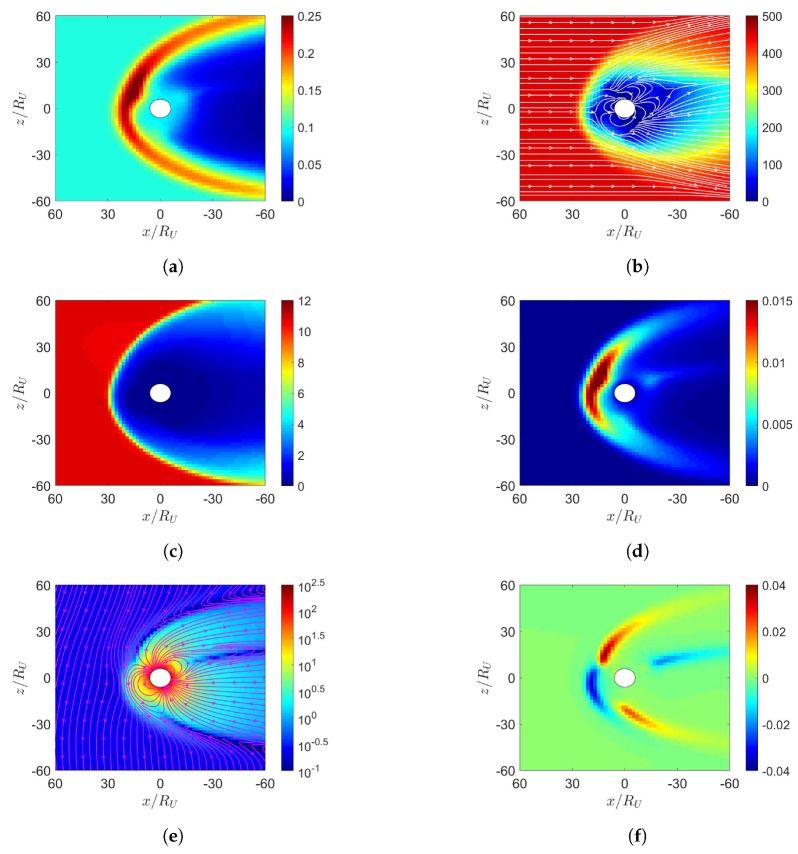
Distributions around Uranus of (**a**) *n* (cm${}^{-3}$), (**b**) ${\bar{u}}_{xz}$ (km/s), (**c**) magnetosonic Mach number, (**d**) *P* (nPa), (**e**) ${\bar{B}}_{xz}$ (nT), (**f**) $J_{y}$ (nA/m${}^{2}$); initial dipole tilts up and towards the sun, southward IMF, t=85.4 min.

Figure 17 shows the simulation results under southward IMF, with the initial dipole tilting up. Figure 17e shows that the planetary magnetic field in the dayside magnetosphere is parallel to the southward IMF, leading to cusp reconnection. The magnetic field in the dayside magnetosphere is compressed by the solar wind to form a closed configuration.

**Figure 18:**
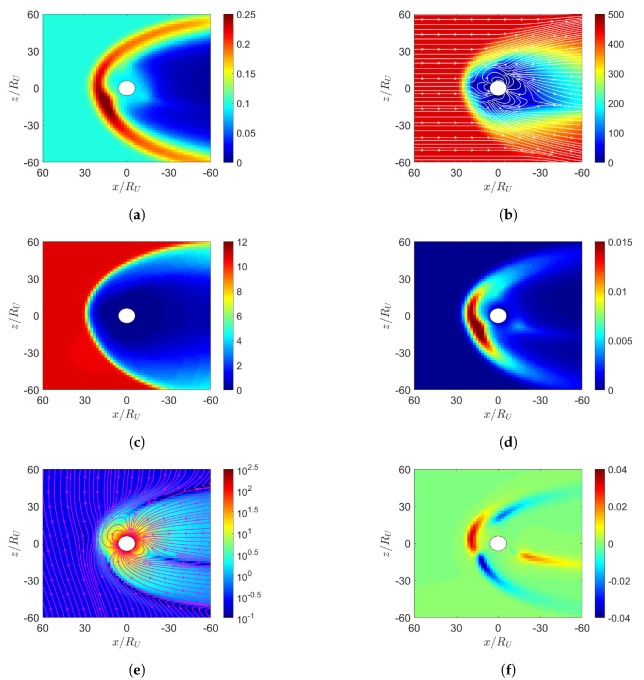
Distributions around Uranus of (**a**) *n* (cm${}^{-3}$), (**b**) ${\bar{u}}_{xz}$ (km/s), (**c**) magnetosonic Mach number, (**d**) *P* (nPa), (**e**) B(nT), (**f**) $J_{y}$ (nA/m${}^{2}$); initial dipole tilts down and towards the sun, northward IMF, t=85.4 min.

Figure 18 shows the simulation results under northward IMF, with the initial dipole tilting down. By comparing Figure 16d and Figure 18d, it is observed that the highest pressure in the dayside magnetosphere appears towards the north and the south magnetic pole, respectively, determined by the pointing direction of the dipole. By comparing Figure 16e and Figure 18e, it is observed that reconnection occurs in the dayside magnetosphere and the cusp region, respectively. Under the same northward IMF, the magnetic field distribution of Uranus gradually evolves from Figure 16e to Figure 18e in half a rotational period of Uranus.

**Figure 19:**
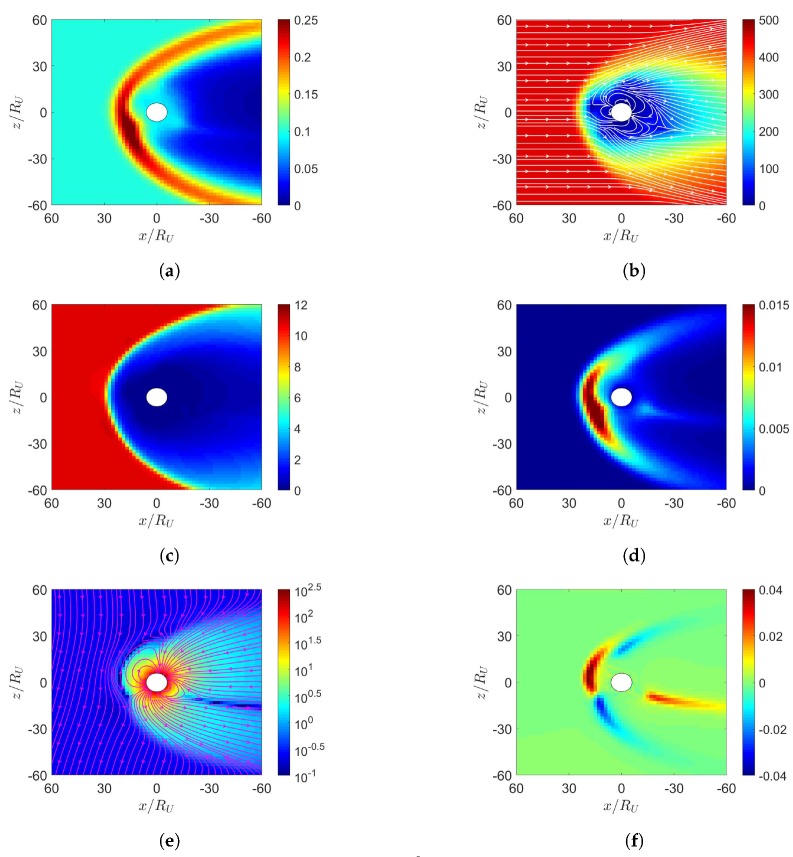
Distributions around Uranus of (**a**) *n* (cm${}^{-3}$), (**b**) ${\bar{u}}_{xz}$ (km/s), (**c**) magnetosonic Mach number, (**d**) *P* (nPa), (**e**) ${\bar{B}}_{xz}$ (nT), (**f**) $J_{y}$ (nA/m${}^{2}$); initial dipole tilts down and towards the sun, southward IMF, t=85.4 min.

Figure 19 shows the simulation results under southward IMF, with the initial dipole tilting down. By comparing Figure 17d and Figure 19d, it is observed that the highest pressure in the dayside magnetosphere appears near the north and the south magnetic pole, respectively, due to different dipole tilt orientations. By comparing Figure 17e and Figure 19e, reconnection appears in the cusp region and the dayside magnetosphere, respectively. Under the same northward IMF, the magnetic field distribution gradually evolves from Figure 17e to Figure 19e in half a rotational period of Uranus.

## 7. Conclusions

MHD simulations have been conducted to investigate the interaction between a northward–southward IMF and the magnetosphere of Mercury, Earth, Jupiter and Uranus, separately. In common, the solar wind induces bow shock in front of the magnetosphere, characterized by high number density and high pressure. The planetary magnetic field and the induced currents in the dayside magnetosphere generate Lorentz force to impede the solar wind. Cusp reconnection is observed when the IMF is parallel to the planetary magnetic field, while dayside reconnection and open magnetic-field configuration are observed when the IMF is antiparallel to the planetary magnetic field.

The magnetosphere of Mercury is relatively small and symmetric about its orbital plane. Under parallel IMF, plasmoid is observed in the magnetotail due to strong solar wind. The magnetosphere of Earth is asymmetric about its orbital plane. The highest pressure in the dayside magnetosphere appears towards the north and south magnetic pole in June and December, respectively. Under southward IMF, plasmoid in the magnetotail lies above the orbital plane in June and below the orbital plane in December. Under northward IMF, the north cusp region shifts leewards in June and windwards in December.

The corotation-enforcing current in the Jovian magnetosphere drags the plasma towards corotation. The outward moving plasma stretches the magnetic field distribution to form a plasmoid in the nightside magnetosphere even under parallel IMF. Under southward IMF, the northern cusp region shifts towards the sun when the dipole tilts towards the sun and shifts away from the sun as the dipole tilts away from the sun. Under northward IMF, the dayside reconnection region shifts southwards of the orbital plane when the dipole tilts towards the sun and shifts northwards of the orbital plane when the dipole tilts away from the sun. Uranus’s magnetosphere gradually evolves from a closed configuration to an open one in half a rotational cycle and vice versa in the next half of a rotational cycle.

## References

[1] Lazio J., Bastian T., Bryden G., Farrell W.M., Griessmeier J.M., Hallinan G., Kasper J., Kuiper T., Lecacheux A., Majid W. (2009). Magnetospheric emissions from extrasolar planets. arXiv.

[2] Russell C.T. (2000). The solar wind interaction with the Earth’s magnetosphere: A tutorial. IEEE Trans. Plasma Sci..

[3] Spohn T., Breuer D., Johnson T. (2014). Encyclopedia of the Solar System.

[4] Ogino T. (1986). A three-dimensional MHD simulation of the interaction of the solar wind with the Earth’s magnetosphere: The generation of field-aligned currents. J. Geophys. Res..

[5] Tanaka T. (1995). Generation mechanisms for magnetosphere-ionosphere current systems deduced from a three-dimensional MHD simulation of the solar wind–magnetosphere-ionosphere coupling processes. J. Geophys. Res. Space Phys..

[6] Wang J., Du A., Zhang Y., Zhang T., Ge Y. (2015). Modeling the Earth’s magnetosphere under the influence of solar wind with due northward IMF by the AMR-CESE-MHD model. China Earth Sci..

[7] Eastwood J.P., Hietala H., Toth G., Phan T.D., Fujimoto M. (2015). What controls the structure and dynamics of Earth’s magnetosphere?. Space Sci. Rev..

[8] Wang Y., Guo X., Tang B.-B., Li W., Wang C. (2018). Modeling the Jovian magnetosphere under an antiparallel interplanetary magnetic field from a global MHD simulation. Earth Planet. Phys..

[9] Cao X., Paty C. (2017). Diurnal and seasonal variability of Uranus’s magnetosphere. J. Geophys. Res. Space Phys..

[10] Tóth G., Kovács D., Hansen K.C., Gombosi T. (2004). Three-dimensional MHD simulations of the magnetosphere of Uranus. J. Geophys. Res. Space Phys..

[11] Goedbloed J.P.H., Poedts S. (2004). Principles of Magnetohydrodynamics: With Applications to Laboratory and Astrophysical Plasmas.

[12] Raeder J., Buchner J., Dum C.T., Scholer M. (2003). Global magnetohydrodynamics—A tutorial. Space Plasma Simulation.

[13] Lyon J., Fedder J., Mobarry C. (2004). The Lyon–Fedder–Mobarry (LFM) global MHD magnetospheric simulation code. J. Atmos. Solar-Terr. Phys..

[14] Toro E.F. (2009). Riemann Solvers and Numerical Methods for Fluid Dynamics—A Practical Introduction.

[15] Gardiner T.A., Stone J.M. (2008). An unsplit Godunov method for ideal MHD via constrained transport in three dimensions. J. Comput. Phys..

[16] Kelley M.C. (2009). The Earth’s Ionosphere: Plasma Physics and Electrodynamics.

[17] Goodman M.L. (1995). A three-dimensional, iterative mapping procedure for the implementation of an ionosphere-magnetosphere anisotropic Ohm’s law boundary condition in global magnetohydrodynamics simulation. Ann. Geophys..

[18] Merkin V.G., Lyon J.G. (2010). Effects of the low-latitude ionospheric boundary condition on the global magnetosphere. J. Geophys. Res..

[19] Brekke A., Moen J. (1993). Observations of high latitude ionospheric conductances. J. Atmos. Terr. Phys..

[20] Jardin S. (2010). Computational Methods in Plasma Physics.

[21] Wang J., Guo Z., Ge Y.S., Du A., Huang C., Qin P. (2018). The responses of the earth’s magnetopause and bow shock to the IMF Bz and the solar wind dynamic pressure: A parametric study using the AMR-CESE-MHD model. J. Space Weather Space Clim..

[22] Yagi M., Seki K., Matsumoto Y. (2009). Development of a magnetohydrodynamic simulation code satisfying the solenoidal magnetic field condition. Comput. Phys. Commun..

[23] Chané E., Saur J., Poedts S. (2013). Modeling Jupiter’s magnetosphere: Influence of the internal sources. J. Geophys. Res. Space Phys..

[24] Wang J., Feng X., Du A., Ge Y. (2014). Modeling the interaction between the solar wind and Saturn’s magnetosphere by the AMR-CESE-MHD method. J. Geophys. Res. Space Phys..

[25] Ioannidis G., Brice N. (1971). Plasma densities in the Jovian magnetosphere: Plasma slingshot or Maxwell demon?. Icarus.

[26] Hill T.W. (1979). Inertial limit on corotation. J. Geophys. Res. Space Phys..

[27] Jia X., Hansen K.C., Gombosi T.I., Kivelson M.G., Tóth G., DeZeeuw D.L., Ridley A.J. (2012). Magnetospheric configuration and dynamics of Saturn’s magnetosphere: A global MHD simulation. J. Geophys. Res. Space Phys..

[28] Kivelson M.G. (2005). The current systems of the Jovian magnetosphere and ionosphere and predictions for Saturn. Space Sci. Rev..

[29] Hill T.W., Dessler A.J., Goertz C.K. (1983). Physics of the Jovian Magnetosphere.

[30] Joy S., Kivelson M.G., Walker R.J., Khurana K.K., Russell C.T., Ogino T. (2002). Probabilistic models of the Jovian magnetopause and bow shock locations. J. Geophys. Res. Space Phys..

[31] Allen R.C., Paranicas C., Bagenal F., Vines S.K., Hamilton D.C., Allegrini F., Clark G., Delamere P.A., Kim T.K., Krimigis S.M. (2019). Energetic Oxygen and Sulfur Charge States in the Outer Jovian Magnetosphere: Insights From the Cassini Jupiter Flyby. Geophys. Res. Lett..

[32] Voight G.-H., Hill T.W., Dessler A.J. (1983). The magnetosphere of Uranus—Plasma sources, convection, and field configuration. Astrophys. J..

